# Cobalt-containing calcium phosphate induces resorption of biomineralized collagen by human osteoclasts

**DOI:** 10.1186/s40824-021-00209-7

**Published:** 2021-03-20

**Authors:** Daniel de Melo Pereira, Matthias Schumacher, Pamela Habibovic

**Affiliations:** Department of Instructive Biomaterials Engineering, MERLN Institute for Technology-Inspired Regenerative Medicine, Maastricht University, Maastricht, Netherlands

**Keywords:** Osteoclastogenesis, Osteoclast resorption, Biomineralized collagen, Intrafibrillar mineral, Bioinorganics, Cobalt

## Abstract

**Background:**

Biomineralized collagen, consisting of fibrillar type-I collagen with embedded hydroxyapatite mineral, is a bone-mimicking material with potential application as a bone graft substitute. Despite the chemical and structural similarity with bone extracellular matrix, no evidence exists so far that biomineralized collagen can be resorbed by osteoclasts. The aim of the current study was to induce resorption of biomineralized collagen by osteoclasts by a two-fold modification: increasing the calcium phosphate content and introducing cobalt ions (Co^2+^), which have been previously shown to stimulate resorptive activity of osteoclasts.

**Methods:**

To this end, we produced biomineralized collagen membranes and coated them with a cobalt-containing calcium phosphate (CoCaP). Human osteoclasts, derived from CD14+ monocytes from peripheral blood, were differentiated directly on the membranes. Upon fluorescent staining of nuclei, F-actin and tartrate-resistant alkaline phosphatase, the cells were analyzed by laser confocal microscopy. Their resorption capacity was assessed by scanning electron microscopy (SEM), as well as indirectly quantified by measuring the release of calcium ions into cell culture medium.

**Results:**

The CoCaP coating increased the mineral content of the membranes by 4 wt.% and their elastic modulus from 1 to 10 MPa. The coated membranes showed a sustained Co^2+^ release in water of about 7 nM per 2 days. In contrast to uncoated membranes, on CoCaP-coated biomineralized collagen membranes, osteoclasts sporadically formed actin rings, and induced formation of resorption lacunae, as observed by SEM and confirmed by increase in Ca^2+^ concentration in cell culture medium. The effect of the CoCaP layer on osteoclast function is thought to be mainly caused by the increase of membrane stiffness, although the effect of Co^2+^, which was released in very low amounts, cannot be fully excluded.

**Conclusions:**

This work shows the potential of this relatively simple approach to induce osteoclast resorption of biomineralized collagen, although the extent of osteoclast resorption was limited, and the method needs further optimization. Moreover, the coating method is suitable for incorporating bioactive ions of interest into biomineralized collagen, which is typically not possible using the common biomineralization methods, such as polymer-induced liquid precursor method.

**Supplementary Information:**

The online version contains supplementary material available at 10.1186/s40824-021-00209-7.

## Introduction

Biomineralized collagen is a promising material in the context of therapies for repair and regeneration of bone defects. It is composed of fibrillar type-I collagen and intrafibrillar mineral in the form of crystallites of calcium phosphate (CaP) of the apatitic phase, oriented with their c-axis parallel to the collagen fibers. As such, biomineralized collagen mimics the composition and structure of bone, at the sub-micron scale, with an unprecedented level of detail [1].

Replicating the structure of bone-like mineralized collagen fibril, and using this unit as a building block for larger constructs, is proposed as a biomimetic method to obtain synthetic bone graft substitutes with load-bearing capacity, as the characteristic structural organization of the collagen and apatite components of the mineralized fibril is one of the major contributors to the mechanical properties of bone [2, 3]. Biomineralized collagen also presents a closer-to-native microenvironment to cells than current bone graft substitutes, which are typically composed of CaP or bioactive glasses [4].

A desirable aspect of a bone graft substitute, besides biocompatibility, is the ability to interact with the microenvironment upon implantation, resulting in direct contact between implanted biomaterial and surrounding bone, thus avoiding undesired fibrous encapsulation. Moreover, biomaterials for bone regeneration should ideally be incorporated in the cellular process of bone remodeling. Biomaterials that can undergo resorption by osteoclasts, the first step in bone remodeling, are gradually replaced by new bone tissue, as osteoclast resorption is followed by deposition of new matrix by osteoblasts [5, 6]. Although bone graft substitutes based on biomineralized collagen closely resemble the physicochemical properties of bone tissue, currently, limited evidence exists that these materials can undergo osteoclast-driven degradation.

In a previous study, we found that osteoclasts were able to form from human monocyte-macrophage precursors on biomineralized collagen membranes; however, they were not capable of efficiently forming sealing zones and resorbing the biomineralized membrane (submitted manuscript). We hypothesized that a combination of material properties was responsible for this lack of active resorption. First, the porous, mesh-like structure of the membranes possibly inhibited the sealing of a compartment for resorption. Second, the membranes exhibited a reduced stiffness when compared to other, frequently used substrates for in vitro study of osteoclast resorption, such as dentin or cortical bone slices, possibly affecting the resorptive activity of osteoclasts. Finally, the absence of native RGD ligands in the membrane (thus not originating from cell culture medium components) to the vitronectin receptor may have been a reason that no osteoclastic resorption of biomineralized collagen membranes occurred [7, 8].

With the goal of triggering osteoclastic resorption, in this study, we propose a modification of a biomineralized collagen membrane by depositing an additional, cobalt-containing CaP coating on its surface. The rationale behind this modification was two-fold. First, by increasing the amount of mineral surrounding the collagen fibers (extrafibrillar mineral), the porosity of the membrane is decreased, possibly enabling osteoclasts to seal a compartment for resorption. Second, the addition of bioactive cobalt ions (Co^2+^) may increase osteoclast activity. Presence of cobalt ions has previously been shown to affect the osteoclast behavior, inducing higher resorptive activity at concentrations around the micromolar range [9].

The addition of trace amounts of Co^2+^ via an extrafibrillar CaP coating is an alternative way of introducing guest inorganic ions in biomaterials based on biomineralized collagen, as cations are usually difficult to incorporate in the intrafibrillar mineral fraction. Indeed, different cations, such as Cu^2+^ and Sr^2+^ have been shown to inhibit the formation of intrafibrillar crystals due to disruption of the polymer-induced liquid precursor (PILP) method, which is commonly used for intrafibrillar mineralization of collagen [10, 11]. More generally, incorporation of small amounts of bioactive inorganic ions is an attractive strategy to affect processes related to bone formation and remodeling, and is interesting also from the perspective of producing closer-to-native materials, as trace amounts of various metallic ions are present in bone apatite [12].

Here we produced and characterized biomineralized collagen membranes modified by deposition of a Co-containing CaP coating, and explored the effect of this modification on the formation and function of osteoclasts derived from human peripheral blood.

## Materials and methods

### Materials

PureCol® collagen type-I solution was purchased from Advanced BioMatrix (San Diego, California, USA, cat#5005). Poly-L-aspartic acid sodium salt (pAsp, Mw = 27 kDa) was purchased from Alamanda Polymers (Huntsville, Alabama, USA, cat#000-D200). 1-Ethyl-3-[3-dimethylaminopropyl] carbodiimide hydrochloride (EDC), N-hydroxysulfosuccinimide (sulfo-NHS), calcium chloride dihydrate, potassium phosphate dibasic, cobalt chloride hexahydrate and all other chemicals were obtained from Sigma-Aldrich (St. Louis, Missouri, USA). Recombinant human M-CSF and soluble RANK-L cytokines were from Peprotech (Connecticut, USA, cat#3002510 and cat#3100110). The β-TCP ceramic discs were kindly supplied by Kuros Biosciences. The β-TCP was prepared as described previously [13], and machined in the shape of discs with a diameter of 9 mm and a thickness of 1 mm.

### Preparation of biomineralized collagen membranes

Biomineralized collagen membranes were prepared as previously described [14, 15]. Briefly, collagen was mixed with 10x concentrated phosphate buffered saline (PBS) and 0.1 M NaOH, in a volume fraction of, respectively: 0,726; 0,246; 0,118. Volumes of 2 mL were gelled in a humidified incubator at 37 °C for 24 h. These gels were then placed on top of a nylon mesh and incubated for 48 h to reduce water content, which resulted in the formation of flat membranes. Next, the membranes were cross-linked overnight at room temperature in 50 mM MES buffer (pH = 7.0), containing 50 mM EDC and 25 mM sulfo-NHS, before proceeding with the intrafibrillar mineralization of the collagen. This was achieved with the PILP method, by incubating the membranes for 7 days at 37 °C in a 50 mM Tris buffer (pH = 7.4) containing 150 mM NaCl, 4.5 mM CaCl_2_, 2.1 mM K_2_HPO_4_ and 100 μg/mL pAsp.

All steps were performed in a sterile environment.

### Cobalt-containing calcium phosphate coating (CoCaP coating)

The already (intrafibrillary) mineralized collagen membranes underwent a two-step coating procedure. In a first step, membranes were incubated in a 2.5x concentrated simulated body fluid (SBF) solution, which was prepared by mixing 3 precursor solutions: buffer, calcium stock and phosphate stock solution, in a ratio of 2:1:1. Buffer solution consisted of 50 mM Tris (pH = 7.4); calcium stock solution consisted of 25 mM CaCl_2_, 1.37 M NaCl and 15 mM MgCl_2_ (in buffer solution); and phosphate stock solution consisted of 11.1 mM Na_2_HPO_4_, 42 mM NaHCO_3_ (in buffer solution). The membranes were placed into 24-well plates, incubated in 2 mL of 2.5x SBF for 3 days at room temperature and finally washed with distilled water. In the second step, the membranes were again placed into 24-well plated and incubated for 3 days at room temperature in 2 mL of a calcium phosphate solution consisting of 50 mM Tris (pH = 7.4), 4 mM CaCl_2_ and 2.25 mM Na_2_HPO_4_, to which a cobalt stock solution of 1 mM CoCl_2_ (in distilled water) was added to achieve final concentrations of 0.1, 1, 5, and 10 μM, respectively. The membranes were then washed with distilled water and prepared for characterization or cell culture. All coating steps were performed in a sterile environment.

### Thermogravimetric analysis

Thermogravimetric analysis was performed on the uncoated and CoCaP-coated biomineralized collagen membranes (*n* = 3). Membranes were washed with MilliQ water and blotted with filter paper before being heated up to 800 °C, at a rate of 5 °C/min, using a Q500 TGA (TA Instruments, Belgium). The leftover weight at 800 °C was considered the mineral content of the membrane.

### Nanoindendation measurements

Nanoindentation measurements were performed in wet state on the uncoated and CoCaP-coated biomineralized collagen membranes, using a Piuma Nanoindenter (Optics11 Life, the Netherlands), which was equipped with a 51.11 k (N/m) probe with a tip radius of 24.5 μm (ref# P190504). Probe calibration was performed using a glass slide submerged in distilled water. Measurements were made in “displacement control” mode, where the tip presses into the substrate until a certain probe displacement is reached (set at 10 μm). An area composed of 4 × 4 points was defined on each membrane, with measurement points (*n* = 16 from one sample) every 200 μm. The Oliver and Pharr method was used to fit the raw data and estimate the elastic modulus. To ensure a flat and stable surface, membranes were bonded to the bottom of a plastic petri dish using double-sided tape, and covered with distilled water.

### Monocyte/macrophage culture and osteoclast differentiation

Poietics^tm^ human peripheral blood CD14+ monocytes were obtained from Lonza (Basel, Switzerland, cat#2 W-400B, lot#647890). Upon thawing the cryovial, cells were transferred to a 15 mL tube containing 9 mL warm basic cell culture medium (α-MEM (Lonza, cat#BE02-002F) supplemented with 10%(v/v) HyClone^tm^ FetalClone^tm^ serum (Thermo Fisher Scientific, USA, cat#10780245, lot#HXSH3008003), 100 U/ml penicillin, 100 μg/ml streptomycin and 20 ng/mL M-CSF), followed by centrifugation at 300 rcf for 5 min. Cells were re-suspended in basic cell culture medium, counted, and seeded at a density of 500.000 cells/cm^2^ on cortical bone slices inside 96-well plates or on the uncoated and CoCaP-coated biomineralized collagen membranes inside 48-well plates. For the first 3 days of culture, the basic medium was modified to contain 35 ng/mL M-CSF. Then, the culture continued in either basic (20 ng/mL M-CSF) or in differentiation medium (basic medium supplemented with 40 ng/mL RANK-L) for up to 21 days, counting from the addition of RANK-L, with medium refreshment every 2–3 days. Before cell seeding, all substrates were incubated for 4 h with basic medium (without M-CSF). All steps were performed in a sterile environment.

### Confocal laser microscopy

Uncoated and coated membranes and cortical bone slices with cells (*n* = 2) were fixed with warm 10% formaldehyde in PBS (15 min at room temperature) for fluorescence staining after 7, 14 or 21 days of culture. The samples were washed three times with PBS, and permeabilized with 0.2% (v/v) Tween-20 in PBS for 10 min at room temperature. For visualization of tartrate-resistant alkaline phosphatase (TRAP) activity, the samples were incubated with ELF-97 phosphate substrate (Thermo Fisher Scientific, USA, cat#E6601) according to the staining method by Filgueira [16]. Briefly, 20x concentrated ELF-97 substrate was diluted in a buffer composed of 110 mM acetate (pH = 5.2), 1.1 mM sodium nitrite and 7.4 mM tartrate, and samples were incubated for 15 min. For staining of F-actin, samples were incubated with 1:200 PBS-diluted AlexaFluor® 488 phalloidin (Thermo Fisher Scientific) for 20 min, and finally for staining nuclei, incubated with 1 μg/mL DAPI (Thermo Fisher Scientific, USA, cat#1306) in PBS for 10 min. All incubation steps were at room temperature, in the dark. Imaging was done with a SP8 STED confocal laser microscope (Leica Microsystems, Germany) using 25x and 86x water-immersion objectives. Large, multinucleated (*n* > 2) and TRAP-positive cells were considered to be osteoclasts. Quantification of the TRAP activity was performed by image analysis using Image J (version 1.52r). Briefly, the number of TRAP-positive pixels was counted for each osteoclast, and expressed as fraction of total cell area. Details of this analysis are provided in a short video as a part of the available dataset published with this study.

### Scanning electron microscopy (SEM)

For SEM morphological characterization, both the uncoated and CoCaP-coated biomineralized collagen membrane were dehydrated in a series of ethanol in water (30, 40, 50, 60, 70, 80, 90, 100%), 15 min per step, followed by 30 min in hexadimethylsiloxane before drying overnight. The membranes with cells underwent the same treatment, but preceded by a cell-lysis step with 1% Triton X-100 in PBS for 30 min in an ultrasonic waterbath, with ice. After dehydration, samples were glued onto aluminum stubs with carbon tape and silver paint, and sputter-coated with a 2 nm layer of iridium using a Q150TES sputter coater (Quorum, United Kingdom). Imaging was performed on a TENEO SEM (FEI, OR, United States) using the T1 in-column or back-scatter detectors in Optiplan mode, with the beam at 2–10 kV and working distance of 2–10 mm.

### Ion-coupled plasma mass spectrometry (ICP-MS)

To determine the total amount of Co^2+^ incorporated in the membranes for different starting concentrations of CoCl_2_ used in the coating solution, membrane samples with a diameter of 6 mm (*n* = 3) were dissolved overnight in 2 mL ultra-pure 60% HNO_3_. Samples were then diluted with MilliQ water to a final concentration of 1% HNO_3_, and 100 μL of diluted sample were mixed with 900 μL of measuring matrix (1% HNO_3_ with 20 ppb scandium). A standard curve of calcium (ranging from 100 to 4000 ppb) and cobalt (ranging from 6 to 200 ppb) was used to quantify the concentrations. Data is presented as molar ratio Co/10^6^ Ca to normalize for the calcium content of each replicate.

For quantification of Co^2+^ release from CoCaP-coated membranes, membrane samples with a diameter of 6 mm (*n* = 3) were incubated with MilliQ water (150 uL in a 96-well plate). Supernatant was collected at designated time-points, stored in micro centrifuge tubes at − 30 °C, and fresh MilliQ was added to the samples. At the end of the experiment, collected supernatants were thawed, and 100 μL sample was added to 900 μL of measuring matrix (1% HNO_3_ with 20 ppb scandium). A standard curve of cobalt (ranging from 6 to 200 ppb) was used to quantify the amount of released ions. Data is presented as the cumulative Co^2+^ concentration over the entire duration of the experiment.

Quantification of Ca^2+^ released during cell culture was performed for cells cultured on either uncoated or CoCaP-coated collagen membranes, in either basic or differentiation medium (*n* = 3 per condition). At every medium refreshment time point, cell culture supernatant was collected in micro centrifuge tubes and the tubes were kept at − 30 °C until collection of all samples was completed. Then, the collected medium was thawed, and 50 μL sample was added to 950 μL of measuring matrix (1% HNO_3_ with 20 ppb scandium). A standard curve of calcium ranging from 100 to 4000 ppb was prepared, and 20 ppb scandium was used as internal standard in all samples measured, to compensate signal drift from the instrument, as well as other factors.

### Statistical analysis

Statistical analysis was performed in GraphPad Prism (version 8.3). The Student’s t-test was used to compare stiffness data from nanoindentation measurements, as well as TGA data. Two-way analysis of variance was used to compare Ca^2+^ concentration data from ICP-MS and TRAP activity in which differences in means between conditions were tested within each timepoint. A Bonferroni post-hoc test was used to correct for multiple comparisons (one family for all comparisons). Mean differences were considered statistically significant for *p*-value < 0.05. In all figures, the following notation applies: * *p* < 0.05; ** *p* < 0.001; *** *p* < 0.0001.

## Results

In this study, we aimed at developing a biomineralized collagen membrane that allows the differentiation of osteoclasts from human peripheral blood monocytes and osteoclastic resorption, by depositing a CoCaP coating on the mineralized collagen fibrils of the membrane.

### CoCaP coating increases stiffness of biomineralized collagen membrane

**Fig. 1:**
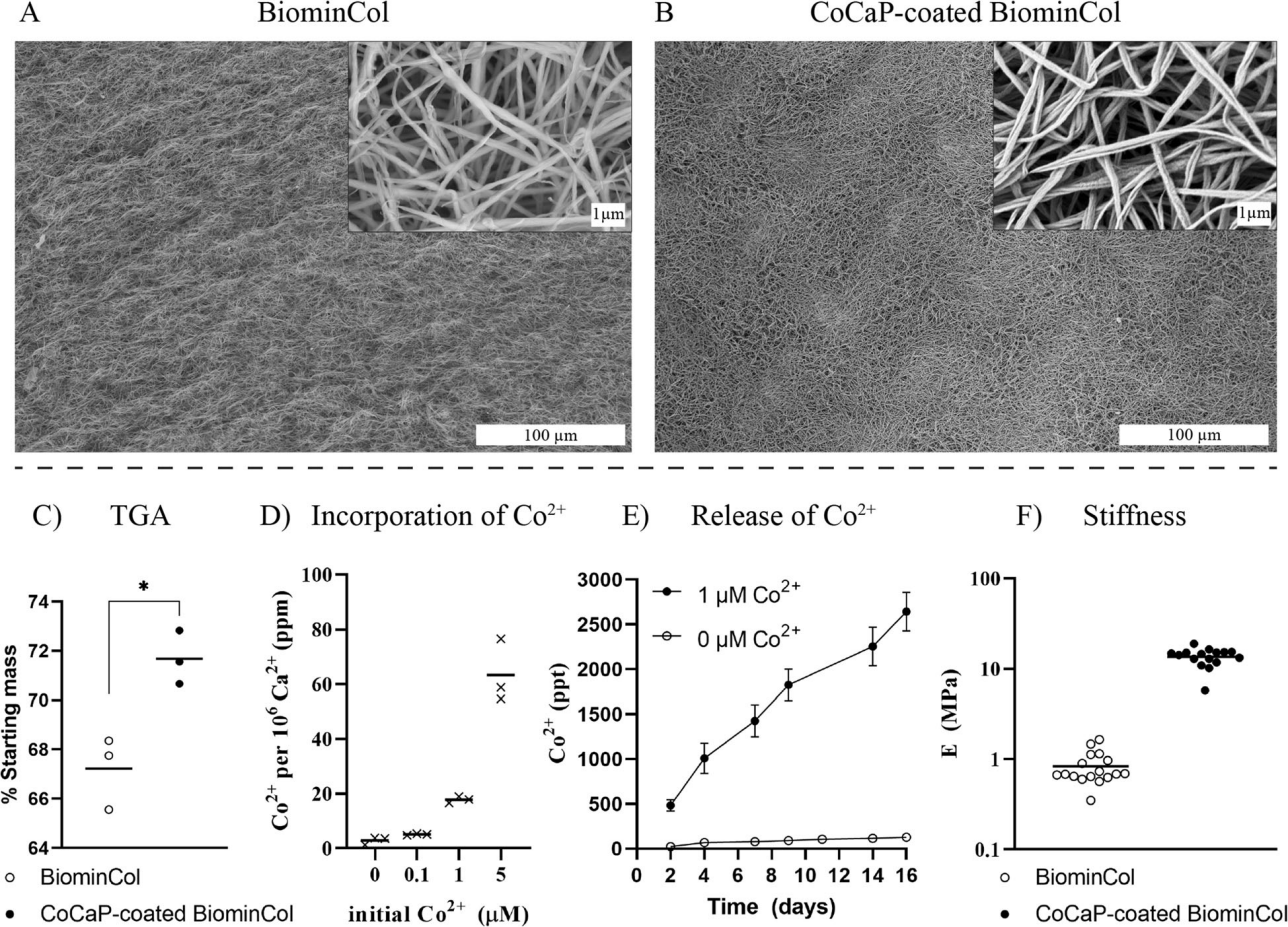
Characterization of uncoated and CoCaP-coated biomineralized collagen (BiominCol) membranes. SEM images of the surface topography of uncoated and CoCaP-coated BiominCol show that both materials have a fibrillar structure. No mineral deposits are seen on the CoCaP-coated BiominCol membrane and the mesh-like structure seems to be preserved after the coating procedure, without obvious differences in fibril diameter. Quantification of Co^2+^ by ICP-MS in CoCaP-coated BiominCol membranes, expressed as the ratio of Co^2+^ to Ca^2+^ for CoCaP coating solutions with different initial Co^2+^ concentrations. The initial concentration of 1 μM was chosen for the rest of the study. Quantification of Co^2+^ released by CoCaP-coated BiominCol membranes in water, expressed as cumulative release over a period of 16 days (*n* = 3). Quantification of mineral content of uncoated and CoCaP-coated BiominCol membranes by TGA, expressed as the remaining mass after burning at 800 °C, showing an about 4% (wt/wt) higher mineral content in CoCaP-coated membranes. Elasticity modulus determined by nanoindentation measurements of uncoated and CoCaP-coated BiominCol. The CoCaP coating increased the membrane modulus of elasticity by about one order of magnitude (*p* < 0.0001)

Biomineralized collagen membranes (BiominCol in figures) were prepared using a modified PILP method as described previously [14, 15]. The membranes had a fibrillar surface structure, as observed by SEM (Fig. 1a). Incubation of the membranes in a CaP solution containing Co^2+^ did not lead to apparent changes of the surface structure, and no mineral deposits were observed (Fig. 1b). TGA results showed that the mass remaining after combustion of the organic phase of the membranes increased from 67% (w/w) for the uncoated collagen membrane to 71% (w/w) for the CoCaP-coated one (Fig. 1c), indicating that the CoCaP coating slightly increased the mineral content of the membrane. The ICP-MS results showed that increasing concentrations of Co^2+^ in the CaP coating solution, caused corresponding increase in the amount of Co^2+^ detected after dissolution of the coated membranes in acid, which confirmed that Co-containing mineral was deposited in the membrane (Fig. 1d). For a starting concentration of 1 μM Co^2+^ there were 20 Co^2+^ per million Ca^2+^. This formulation was chosen for all other experiments in this study. CoCaP-coated collagen membranes showed a continuous release of Co^2+^ in water over a period of 16 days (Fig. 1e), with an average release of approximately 400 ppt per refreshment period (2–3 days), which is equivalent to a molarity of around 7 nM. Nanoindentation measurements further showed that the stiffness of the CoCaP-coated collagen membrane (about 10 MPa) was about one order of magnitude higher relative to the uncoated membrane (Fig. 1f), which is likely a result of the presence of additional mineral.

### Osteoclasts differentiate from monocytes and show actin rings on CoCaP-coated mineralized collagen membranes, but not on uncoated membranes

**Fig. 2:**
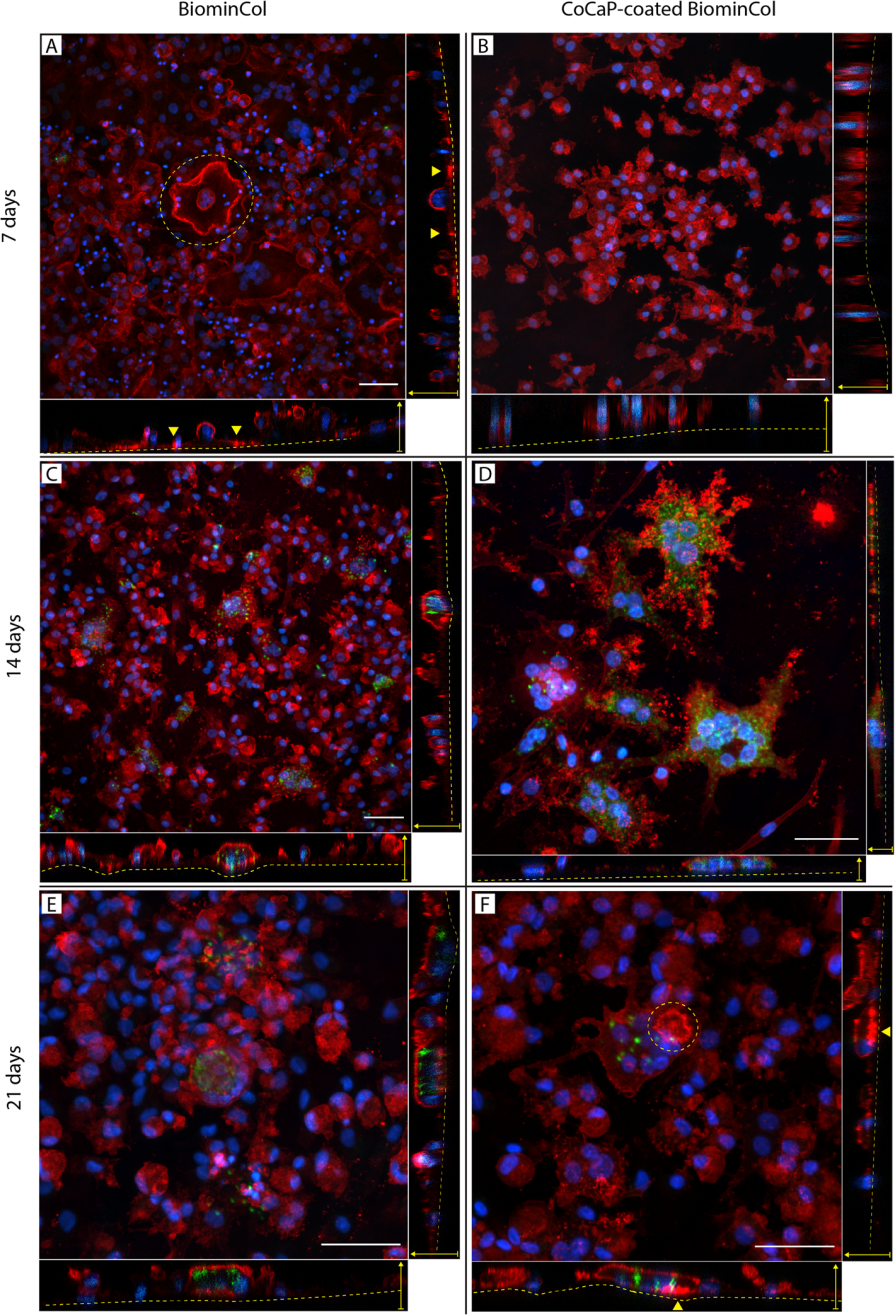
Confocal laser microscopy images (top and side view) of osteoclasts cultured on uncoated (**a**, **c**, **e**) and CoCaP-coated BiominCol membranes (**b**, **d**, **f**), at 7, 14 and 21 days. At 7 days, multi-nucleated cells were visible on uncoated BiominCol, with the central cell showing a thick actin structure (podosome belt - dashed circle). CoCaP-coated BiominCol membrane also exhibited multi-nucleated cells at this point. No TRAP+ cells were observed on either substrate. At 14 days, several TRAP+ multi-nucleated large cells were observed on both surfaces. High intensity actin structures were observed close to the surface of the material, but they do not appear to be actin rings. At 21 days, actin structures resembling actin rings were observed on some osteoclasts on CoCaP-coated BiominCol membranes (dashed circle and arrowheads), but not on the uncoated ones. Dashed lines in the side views represent the surface of the membrane. For all images: scale bar is 50 μm, actin in red, nuclei in blue, and TRAP in green

**Fig. 3:**
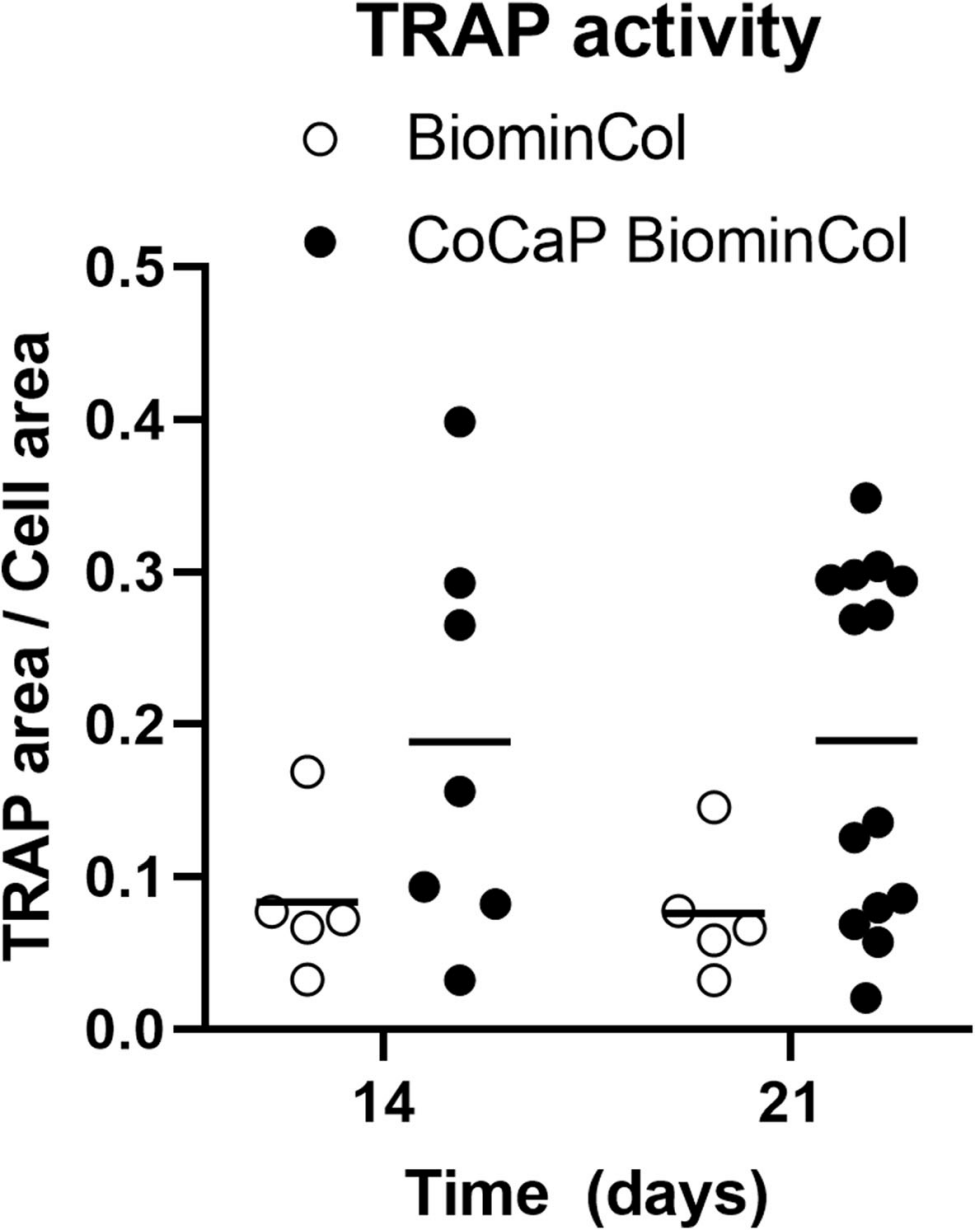
Semi-quantitative comparison of TRAP activity between osteoclasts cultured on BiominCol and CoCaP-coated BiominCol. TRAP activity is shown as the area fraction of TRAP+ pixels relative to the whole cell area, for each osteoclast (each point represents one osteoclast)

Monocytes derived from human peripheral blood were cultured on uncoated and CoCaP-coated biomineralized collagen membranes, and stimulated to differentiate into osteoclasts by the addition of 40 ng/mL RANK-L to basic cell culture medium. After 7 days in differentiation medium, a few large and multinucleated cells were observed on the uncoated membranes (Fig. 2a), but not on CoCaP-coated membranes (Fig. 2b). TRAP-positive cells were not observed on either of the membranes after 7 days, but were present on both membranes after 14 and 21 days. Quantification of TRAP activity by image analysis showed that on average, TRAP activity of osteoclasts on CoCaP-coated membranes was twice as high as that on uncoated membranes at 14 and 21 days (*p* = 0.01) (Fig. 3). After 14 days of culture, osteoclasts on both uncoated and coated membrane showed concentrated actin structures (Fig. 2c-d). These actin structures were dispersed through the cell and did not form clear actin rings. After 21 days of culture in differentiation medium, a few osteoclasts on the CoCaP-coated membranes exhibited circular, high-intensity actin structures that resemble actin rings (Fig. 2f). It should however be noted that the number of osteoclast with clearly defined actin rings was limited. These structures were not detected on the uncoated biomineralized collagen membrane (Fig. 2e).

### Presence of co and increased stiffness stimulate limited resorption of the coated biomineralized collagen membrane

**Fig. 4:**
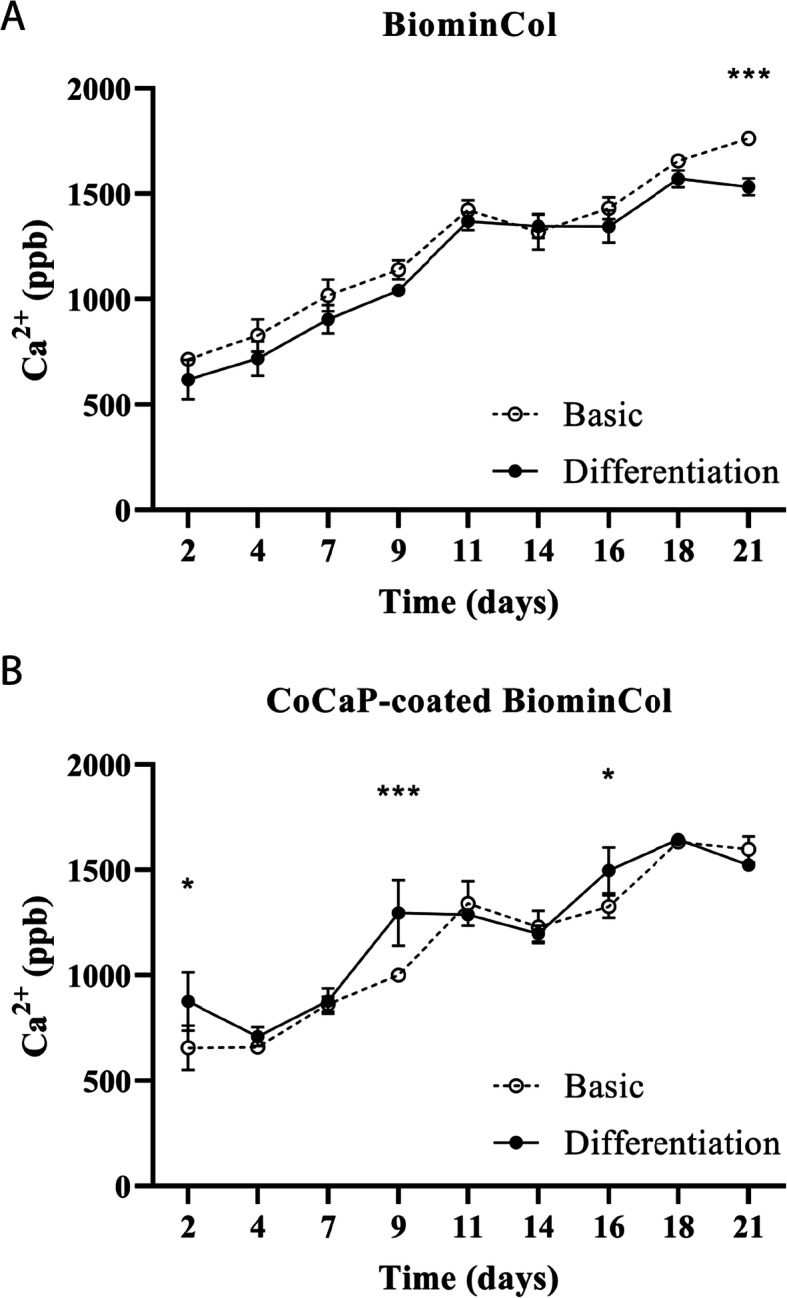
Quantification of Ca^2+^ in cell culture medium by ICP-MS over a period of 21 days of cell culture in basic or differentiation medium. **a** For uncoated BiominCol membranes, no major differences between the basic and differentiation medium were observed, indicating that resorption by osteoclasts was limited or non-existent. **b** For CoCaP-coated BiominCol membranes, a significantly higher Ca^2+^ concentration was seen for the differentiation condition on day 2 (*p* = 0.37), day 9 (*p* < 0.001) and day 16 (*p* = 0.016)

Resorption of uncoated and CoCaP-coated biomineralized collagen membranes was evaluated by measuring the Ca^2+^ concentration in the cell culture medium, up to 21 days (Fig. 4). This method allows for indirect quantification of resorptive activity, and is suitable for materials for which quantification of resorbed area or volume by conventional imaging methods is difficult, for example because of the high roughness or porosity of the substrate as is the case for collagen membranes. Nevertheless, a limitation of this method is a relatively low sensitivity; as the Ca^2+^ concentration in medium varies with many processes associated with cell culture, only major differences in resorptive behavior can be quantified. For example, when biomineralized collagen is immersed in cell culture medium in the absence of cells, there is a depletion of Ca^2+^ from the medium (data not shown). Nevertheless, the substrate-based effects are accounted for in the results as a comparison is only made between cells cultured in basic versus differentiation medium, on the same substrate. Therefore, any difference in Ca^2+^ concentration should be a result of cell differentiation and resorptive activity.

On the uncoated membranes, no significant differences in Ca^2+^ concentration were observed between cultures in basic and differentiation medium, except at the last time point of 21 days, when there was a significantly higher Ca^2+^ content in the basic medium condition (Fig. 4a). This is an unexpected finding, as no Ca^2+^ release should occur from the substrate in the absence of cells, or from macrophage activity. On the CoCaP-coated membrane, there were significant differences in Ca^2+^ concentration between basic and differentiation medium at 2, 9 and 16 days, with differentiation medium having a higher Ca^2+^ content (Fig. 4b).

**Fig. 5:**
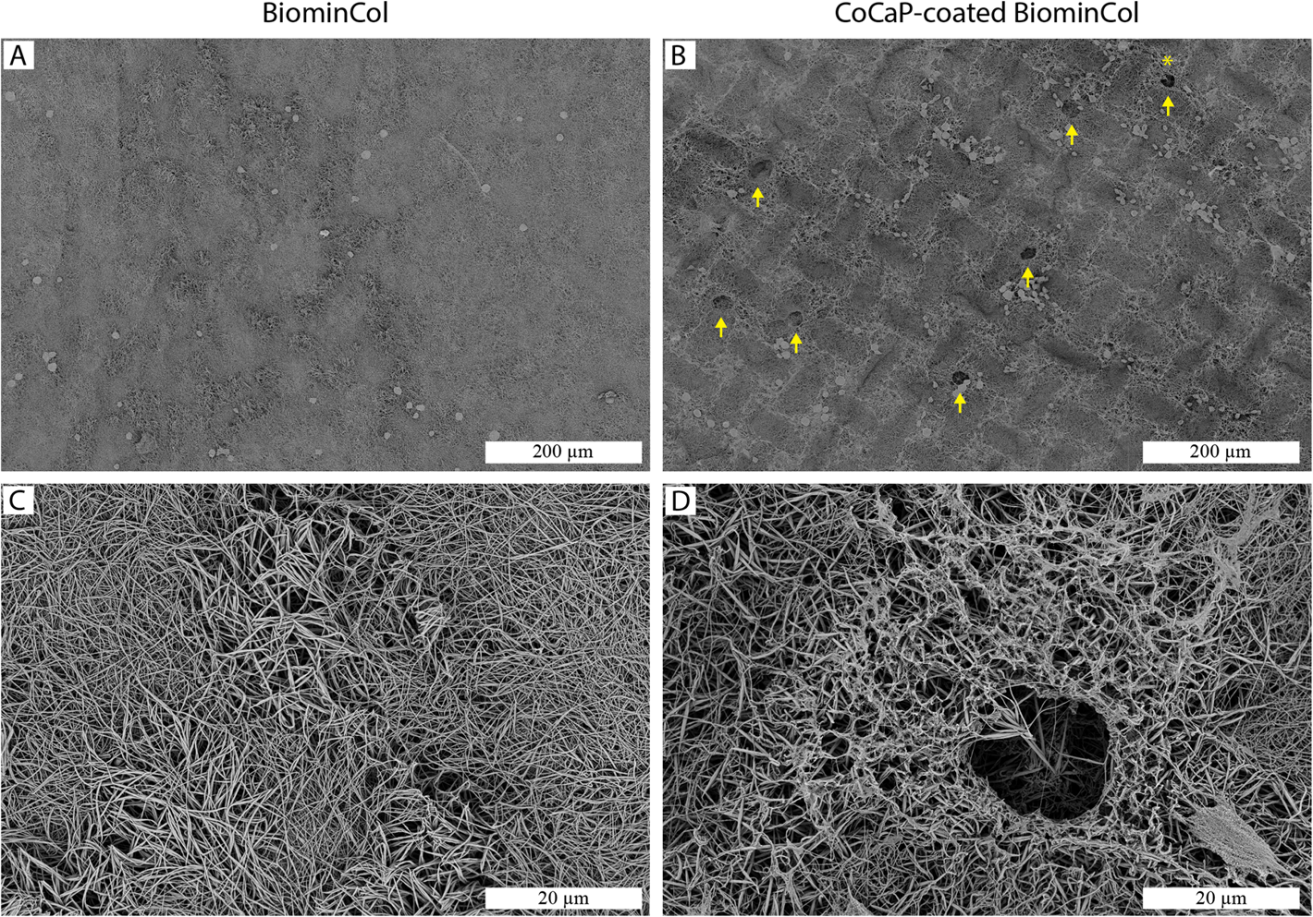
SEM images of uncoated (**a**, **c**) and CoCaP-coated BiominCol membranes (**b**, **d**) after 21 days of cell culture. Cells were removed prior to imaging. Both substrates showed signs of disruption of intrafibrillar membrane structure by cells. On the CoCaP-coated BiominCol membrane, rounded depressions with a diameter of about 20 μm were observed that resemble resorption lacunae (yellow arrows)

To further investigate whether the differences in Ca^2+^ concentration were due to osteoclast resorption, the same uncoated and CoCaP-coated membranes for which the ICP-MS analysis of the medium was performed, were observed by SEM, after removing the cells. On uncoated biomineralized collagen membranes, no obvious signs of resorption were observed (Fig. 5a). There were areas on the membrane where the fibrillar structure appeared to be disturbed, which probably correspond to places of cell attachment (Fig. 5c). On the CoCaP-coated membranes, the same areas with disrupted fibrillar structure were also present, but interestingly, some of these areas also exhibited resorption lacunae-like features, i.e., pockets indented in the surface of the biomaterial (Fig. 5b, d).

## Discussion

### CoCaP coating increases stiffness of biomineralized collagen membrane

The modified PILP method, followed by a coating method was used to develop CoCaP-coated biomineralized collagen membrane. The absence of apparent mineral deposits on the surface (Fig. 1b) after incubation of the biomineralized collagen membrane in the CoCaP solution was unexpected, as the TGA data showed a 4% (w/w) increase in mineral content after the coating process (Fig. 1c). Moreover, previous studies have shown that CaP nodules (spherulitic mineral aggregates) were deposited on tissue culture plastic upon immersion in CaP solutions similar to the one used here, with or without Co^2+^ [17, 18]. It has also been shown that CaP mineral deposits formed on collagen scaffolds upon incubation into concentrated SBF solutions [19–21].

A few aspects related to the properties of the biomineralized collagen membrane and the CaP coating method used here may explain why this extra mineral deposition, originating from the coating, was not observed on SEM images. First, the surface area of the mineralized collagen membranes was much higher than that of cell culture plates used in the earlier studies [17, 18], which means that the same amount of mineral that would normally deposit on a flat surface could now form on many more available nucleation/growth sites, resulting in the formation of smaller mineral particles. Second, the CoCaP coating step provided less total mineral than, for example, the PILP method used for intrafibrillar collagen mineralization; the CoCaP coating solution had about ten times lower ion content than the PILP solution, due to the lower reaction volume used, as described in the methods section. This may explain why there was no noticeable fiber diameter increase after the coating step (uncoated BiominCol at 188 ± 45 nm and CoCaP-coated BiominCol at 156 ± 30, *n* = 20), in contrast to the observed fiber diameter increase following the intrafibrillar mineralization [14, 15]. Finally, and perhaps most importantly, the biomineralized collagen membranes used in our study already contained intrafibrillar CaP mineral before immersion into the CoCaP coating solution, which may have interfered with nucleation and growth of CaP deposits on the fibrous collagen surface. It is suggested that CoCaP coating preferentially crystallized on the pre-existing intrafibrillar CaP. This seems plausible based on earlier studies that compared the rate of hydroxyapatite (HA) crystal growth on collagen, elastin and HA surfaces. These studies showed that the growth rate of HA on the HA surface was several orders of magnitude higher than on the polymeric substrates [22–24]. It should be noted that we tried to incorporate Co^2+^ intrafibrillarly, by adding the ion to the PILP solution. This was, however, unsuccessful as no intrafibrillar mineral was formed. This result is in accordance with previous reports in which inhibitory effects of cations such as Cu^2+^ [10] and Sr^2+^ [11] on the PILP-induced biomineralization were observed.

Taken together, based on the SEM characterization of the structural properties of mineralized collagen membranes before and after immersion in the CoCaP coating solution, there was no evidence for the deposition of the mineral on the surface; however, it is suggested that the new mineral deposited on the pre-existing intrafibrillar mineral, which could not be observed using SEM imaging. This hypothesis was supported by the TGA data, presented above, as well by the results of the ICP-MS analysis of the Co^2+^ content of coated membranes, measured for different initial concentrations of Co^2+^ in the solution and presented as the number of Co^2+^ per 10^6^ Ca^2+^ (Fig. 1d).

The additional 4% (w/w) mineral content, formed during the coating process, is likely responsible for the increased stiffness of the CoCaP-coated collagen membrane (Fig. 1f). It should be noted that both the uncoated and CoCaP-coated collagen membranes prepared here were significantly less stiff than the biomineralized collagen membrane reported in an earlier study (177 MPa) [15]. Although a similar biomineralization method was used in both studies, differences in fiber density and density of crosslinks between the materials obtained in the two studies may explain different mechanical properties.

The TGA, ICP-MS, and nanoindentation data showed that the CoCaP coating process changed the physicochemical properties of the substrate, by incorporation of Co in its composition and by increasing its stiffness, without significantly altering the surface morphology of the biomineralized collagen membrane.

### Osteoclasts differentiate from monocytes and show actin rings on CoCaP-coated mineralized collagen membranes, but not on uncoated membranes

No multinucleated TRAP+ cells were observed on either type of membrane at day 7, but at day 14 and 21, osteoclasts were observed on both uncoated and CoCaP-coated biomineralized collagen membranes (Fig. 2c-f), and TRAP activity was on average twice as high on the CoCaP-coated membranes (Fig. 3). Moreover, osteoclasts showing actin rings were only observed on the CoCaP-coated membranes (Fig. 2f). Intracellular TRAP activity was previously shown to correlate with resorption of CaP cements, while correlation between extracellular TRAP activity and resorption was reported for dentin and osteoblast-produced matrix [25]. Further research is needed to investigate whether the more pronounced TRAP activity on CoCaP-coated mineralized collagen membranes observed here is indicative of the coated membranes being resorbed by osteoclasts.

In an earlier study, we have shown that biomineralized collagen membranes supported osteoclast formation. Nevertheless, the formed osteoclasts were unable to form stable actin rings or sealing zones, and were therefore incapable of resorption. Here, we observed for the first time osteoclasts with actin rings on the CoCaP-coated biomineralized collagen membranes. As was described in the previous section, the coated membranes contained additional CaP with Co^2+^ and had increased stiffness relative to the uncoated ones. Both modifications may have contributed to the difference in osteoclast phenotype, i.e., the capacity to form actin rings, but it is likely that the increased stiffness is the main factor. Few studies investigated the effects of Co^2+^ on formation and resorptive activity of osteoclasts in the similar concentration range as used here. In a study by Patntirapong et al., surface of tissue culture well plates was coated with thin CaP layers containing Co^2+^ using solutions with a Co^2+^ concentration of 0.1, 1, and 5 μM, respectively. These coatings, which were shown to release about 1, 10, and 50 ng of Co^2+^ in 500 μL cell culture medium, respectively over a period of 3 days, supported the formation of a higher number of murine osteoclasts (1 μM condition in particular), and larger resorbed areas (all three conditions) [9]. For comparison, after 2-day incubation in water of the CoCaP-coated collagen membrane used in our study, the Co^2+^ concentration was around 0.4 μg/L (equivalent to around 7 nM), which is lower than the Co^2+^ released in the 0.1 μM condition of the above discussed study (2 μg/L). Another study showed that 10 nM of dissolved Co^2+^ had either no effect on the resorptive activity of forming human osteoclasts, or caused a slight drop in the resorptive activity of mature osteoclasts [26]. Taken together, the CoCaP-coated collagen membranes used in our study contained a lower amount of Co^2+^ than the studies in which a stimulatory effect on differentiation and resorptive activity of osteoclasts was observed. Nevertheless, differences in experimental set up (e.g. cell type) do not allow a direct comparison and therefore, an effect of Co^2+^ ions on osteoclast formation observed in our study cannot be fully excluded.

While low doses of Co^2+^ seem to stimulate osteoclast resorptive activity, there is no information on whether they play a role in actin ring formation. Substrate stiffness, on the other hand, has been suggested to have an influence on actin ring formation. In a study where the formation of podosomes by osteoclasts cultured on polyacrylamide and polydimethylsiloxane surfaces ranging in stiffness from 30 kPa to around 1800 kPa was investigated, it was shown that podosome belt formation was possible in this range of stiffness, as long as integrins were activated (e.g., by coating the substrate surfaces with either collagen or vitronectin) [27]. These findings suggested that actin ring formation can occur, to a certain extent, independently of substrate stiffness, if all other conditions (e.g. integrin activation) are met. In another study, it was shown that osteoclasts were also able to form actin rings on collagen-coated coverslips, but not on the surface of collagen gels [28]. This study suggested that the mechanical properties and/or the porous, mesh-like surface of the gel impeded actin ring formation.

In our study, we have shown that biomineralized collagen, with stiffness of around 1000 kPa, did not allow actin ring formation on its surface. Since biomineralized collagen contains ligands for β_1_ integrin activation (but not ligands for the vitronectin receptor), and stiffness within the range that allows actin ring formation, it is suggested that the porous structure was the inhibiting factor for actin ring formation. However, when stiffness was increased up to 10 MPa by coating the membrane with CoCaP, actin rings were sporadically observed, which suggests that the inhibiting effect of the fibrillar surface on actin ring formation can be at least partially overcome by increasing the substrate stiffness.

### Presence of co and increased stiffness stimulate limited resorption of the coated biomineralized collagen membrane

Resorption of the biomineralized membranes was investigated by quantification of Ca^2+^ in cell culture medium by ICP-MS, and also by observation of the membranes by SEM, after cell removal. Higher Ca^2+^ concentration was detected in culture medium from CoCaP-coated membranes at 2, 9 and 16 days. While it is unlikely that resorption occurred as early as on day 2, as osteoclasts were not observed on the coated membrane even after 7 days (Fig. 2), it is plausible that the higher Ca^2+^ content in differentiation medium at 9 and 16 days was due to resorption of the substrate. However, these results must be interpreted with caution, due to the fact that only a few resorbing osteoclasts were observed and that this technique is not optimal for determining small differences in osteoclastic resorption.

There were no signs of resorption on the uncoated membranes (Fig. 5a, c), but resorption lacunae were observed on the CoCaP-coated membrane (Fig. 5b, d). These were of the same size as the actin rings observed by confocal laser microscopy. The observation of actin rings by fluorescence microscopy, together with SEM observations of resorption lacunae, and increased Ca^2+^ in cell culture medium after 9 and 16 days all point towards events of osteoclast resorption taking place on the CoCaP-coated biomineralized membrane, although to a limited extent. Another interesting observation was that all resorption lacunae observed using SEM had a pit-like structure, while no trench-like resorption lacunae were observed.

In the previous section, we discussed the influence of substrate stiffness on actin ring formation by osteoclasts on different types of substrate. To our knowledge, no studies exist that investigated the effect of stiffness on osteoclastic resorption, especially in the range of kPa to MPa, which would be useful to compare to our own data. From various resorption studies on bone, dentine, or CaP ceramics, it is evident that in-vitro resorption on surfaces with higher stiffness is common [29–31]. We have also performed differentiation and resorption experiments on porous β-TCP ceramic discs to confirm that the differentiated osteoclasts were able to resorb this substrate (Supplementary Figure 1). The stiffness of bone, dentine and CaP ceramics is in the GPa range, thus orders of magnitude higher than the biomineralized collage substrates used in our study. Although resorption studies on polymer-CaP composites exist [32–34], and these materials can have lower stiffness, the resorption results in these studies are commonly not discussed in the context of the mechanical properties of the substrate.

Taken together, the results of this study showed that coating a biomineralized collagen membrane with a layer of Co-containing CaP had a twofold effect on the membrane properties, i.e. the stiffness of the membrane was increased by an order of magnitude, to 10 MPa, and Co was added to the substrate (Fig. 1d, f). Additional CaP (Fig. 1c) that was added by the coating process was involved in both effects by, on the one hand stiffening the membrane and on the other acting as a carrier of Co. Interestingly, no obvious effects of the additional coating was observed on the fibril thickness and hence the porosity of the membrane (Fig. 1a, b). Osteoclasts, differentiated from human peripheral blood monocytes, were able to form on the coated membranes, and were also capable of resorbing it, albeit to a limited extent. This is supported by the observation of actin ring formation using fluorescence microscopy (Fig. 2f), as well as by the observation of pit-like formations on the membranes, using SEM after cell removal (Fig. 5b, d). The higher concentration of Ca^2+^ in cell culture medium of differentiated cells at days 9 and 16 also support the few, and sporadic resorption events observed (Fig. 4b). Although the effect of Co^2+^ ions, which were present at a low concentration (Fig. 1d), cannot be excluded, we hypothesize that the main contributor to the ability of osteoclasts to resorb the coated membrane was the increase in membrane stiffness (though still significantly lower than that of bone or dentin slices), that allowed sealing of a compartment for resorption, partly overcoming the resorption-inhibiting effect of the large pores.

The main aim of this study was to modify the biomineralized collagen membrane in such a way that it can be resorbed by osteoclasts by adding Co^2+^ ions to the material. As discussed above, since the method we used to achieve this changed more than only the chemical properties of the membrane, the limitation of this study is that we cannot conclude which material property was responsible for the observed biological response. To this end, additional experiments separating the effects of stiffness and chemistry are required.

## Conclusion

In conclusion, the results of this study showed that by a relatively simple modification of a biomineralized collagen membrane, resulting in increased stiffness and addition of a bioactive ion, it was possible to trigger the osteoclastic resorption of the membrane, though to a limited extent. The results of this study provide some directions for continued improvement of biomineralized collagen materials for application as bone graft substitutes. First, it is suggested that increase in collagen membrane stiffness enhances osteoclastic resorption. This can be achieved by compacting the collagen before intrafibrillar mineralization, or by increasing the amount of extrafibrillar mineral surrounding the already-mineralized fibers. The latter would have the same effect as reducing the pore size by compacting, which is an increase in stiffness, likely resulting in a surface more amenable to osteoclastic resorption. Secondly, the method of introducing bioactive ions to the membranes by post modification as described here offers an attractive alternative to incorporation during the intrafibrillar mineralization with the PILP method, which is complex, and should be explored more extensively for different types of ions.

## Supplementary Information

**Additional file 1: Supplementary Figure 1**. Confocal laser microscopy (A, B) and SEM (C-F) images of osteoclasts culture on β-TCP discs, after 21-day cell culture. Laser confocal microscopy image (A) showed actin structures resembling resorption rings in close contact with the substrate surface (scale bar 50 μm). These are more clearly visible in the grayscale actin image (B), taken from a Z position close to the surface of the ceramic disc. SEM image of a large cell (C), presumably an osteoclast. Morphology of resorption pit (C-F) at different magnifications. The interior of the pit (F) showed β-TCP grains with etched surface.

## Data Availability

The dataset supporting the conclusions of this article is available in the Dataverse repository, 10.34894/JUS4U6. This link will become publicly available after publication, in the meantime the dataset can be accessed from this private link, https://dataverse.nl/privateurl.xhtml?token=c386c284-9ffd-4003-a853-04caba088cd4.
