# Supplementary figures and images for: Cobalt-containing calcium phosphate induces resorption of biomineralized collagen by human osteoclasts
Source: Biomater Res. 2021 Mar 20;25:6. doi: 10.1186/s40824-021-00209-7 (PMC7981861; doi:10.1186/s40824-021-00209-7)

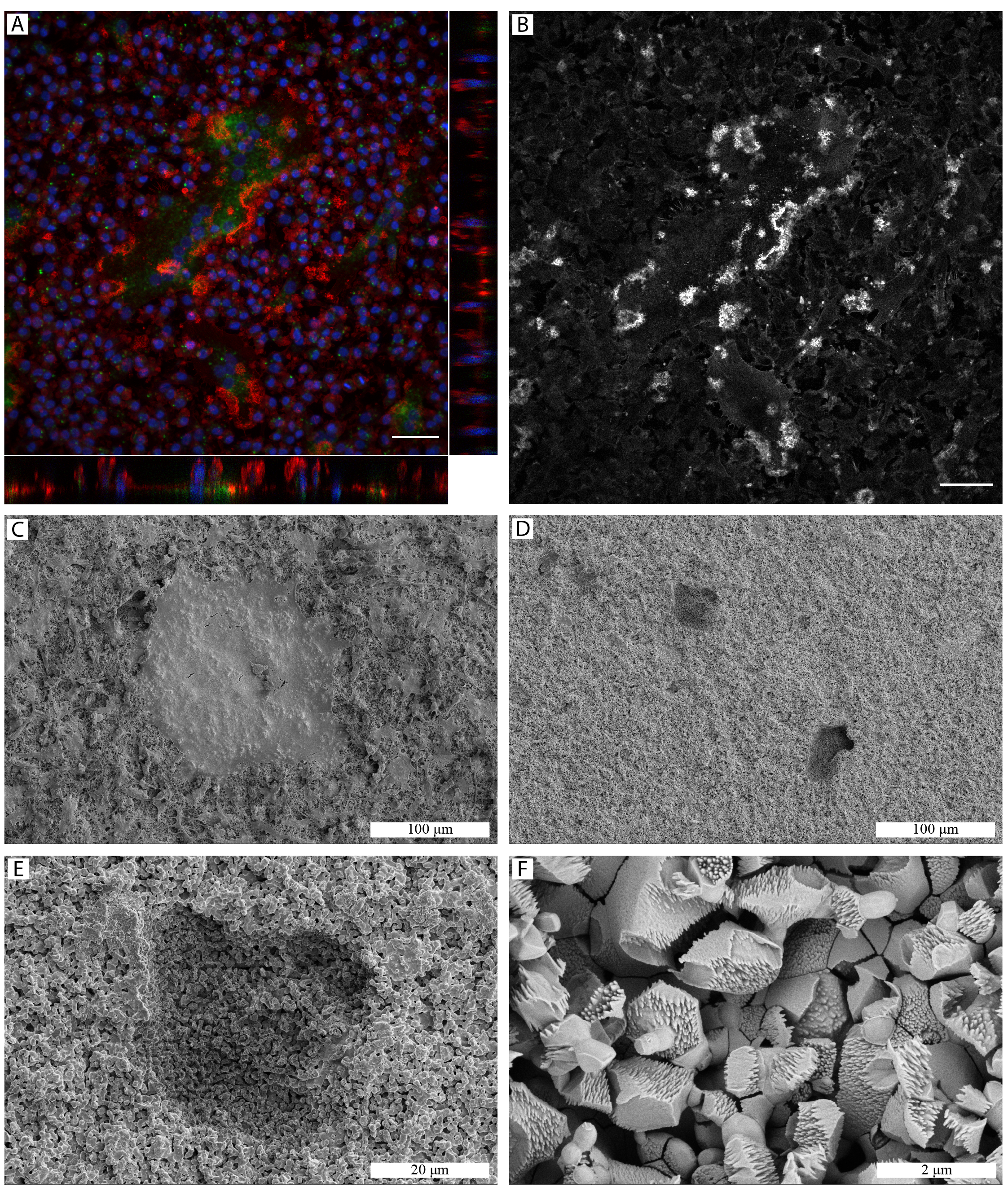

Supplement: Supplementary file 1 — Additional file 1: Supplementary Figure 1. Confocal laser microscopy (A, B) and SEM (C-F) images of osteoclasts culture on β-TCP discs, after 21-day cell culture. Laser confocal microscopy image (A) showed actin structures resembling resorption rings in close contact with the substrate surface (scale bar 50 μm). These are more clearly visible in the grayscale actin image (B), taken from a Z position close to the surface of the ceramic disc. SEM image of a large cell (C), presumably an osteoclast. Morphology of resorption pit (C-F) at different magnifications. The interior of the pit (F) showed β-TCP grains with etched surface. [file 40824_2021_209_MOESM1_ESM.tif]
